# Changes in practice of less‐invasive surfactant administration (LISA) in United Kingdom neonatal units

**DOI:** 10.1111/apa.17446

**Published:** 2024-10-08

**Authors:** Sandeep Shetty, Donna Tolentino, Cheryl Lau, Donovan Duffy, Anne Greenough

**Affiliations:** ^1^ Neonatal Intensive Care Centre St George's University Hospitals NHS Foundation Trust London UK; ^2^ St George's University of London London UK; ^3^ Department of Women and Children's Health, Faculty of Life Sciences and Medicine, School of Life Course Sciences King's College London London UK

**Keywords:** less‐invasive surfactant administration, surfactant administration

## Abstract

**Aim:**

To determine whether the use of less‐invasive surfactant administration (LISA) had changed between 2018 and 2024.

**Methods:**

An online questionnaire was sent to all 191 neonatal units between June 2023 and May 2024. One consultant from each neonatal unit was randomly selected. Follow‐up was done by telephone (middle‐grade doctor grade and above or alternatively to Advanced Neonatal Nurse Practitioners) for the non‐responders.

**Results:**

Response rate was 100%from 191 units neonatal units. LISA was used in 134 (70%) neonatal units in 2024 compared to 35 (18.7%) units in 2018 (*p* < 0.001). The reason why LISA was not performed was lack of experience/training (51%) or not having a standardised practice/guideline (49%). LISA in the delivery suite (DS) had increased from 2% in 2018 to 16% in 2024, and the use of video laryngoscope for LISA is becoming standard of practice. The oxygen requirement criteria for the use of LISA in both the DS and on neonatal unit had reduced to FiO2 of 0.3 or more.

**Conclusion:**

The uptake of LISA had increased in the United Kingdom. There is greater use of LISA in the DS. Lack of training and expertise were the major limiting factors for LISA not being performed.

**Abbreviations**
ANNPs: advanced nurse practitioners
BPD: Bronchopulmonary Dysplasia
CPAP: continuous positive airway pressure
DS: delivery suite
FiO_2_
: inspired fraction of oxygen
GA: gestational age
INSURE: INtubation‐Surfactant‐Extubation
LISA: less‐invasive surfactant administration
LNU: local neonatal units
NICU: neonatal intensive care units
NIV: non‐invasive ventilation
NNU: neonatal unit
NONA‐LISA: NON‐pharmacological Approach Less Invasive Surfactant Administration
PRELISA: Premedication for Less‐Invasive Surfactant Administration Study
RCT: randomised control trial
RDS: respiratory distress syndrome
SCBU: special care baby units
UK: United Kingdom
VDL: Video laryngoscope

**Key Notes**

- This survey highlights the change in practice for less‐invasive surfactant administration (LISA) in United Kingdom neonatal units.
- The uptake of LISA has increased in the United Kingdom with more units offering LISA in delivery suite as standard of practice.
- Lack of training and expertise were the major limiting factors for neonatal units not performing LISA.

## 1 INTRODUCTION

During less‐invasive surfactant administration (LISA), surfactant is delivered directly into the lungs via a fine‐bore catheter inserted into the trachea.^1^ The European Consensus Guidelines on the management of respiratory distress syndrome (RDS) state that LISA rather than INSURE (INtubation‐Surfactant‐Extubation) is the preferred mode of surfactant administration for spontaneously breathing preterm babies supported by continuous positive airway pressure (CPAP).^1^ LISA has been widely adopted in many parts of Europe and, in large cohort studies, there are better clinical outcomes.^1^, ^2^, ^3^ Two‐year follow‐up from one of the larger randomised trials gave reassurance that the LISA technique is safe.^4^ The largest study (OPTIMIST) randomised 485 babies born between 25 and 28 weeks of gestation: LISA versus sham procedure at an inspired fraction of oxygen (FiO_2_) threshold of 30%. Although there was no significant difference in the primary outcome of death or bronchopulmonary dysplasia (BPD), there was a significant reduction in BPD in survivors favouring the treated infants (37% vs. 45%).^5^

A survey in 2017 with a 51% response rate was demonstrated. LISA was being used in 48% of European unit.^6^ A United Kingdom (UK) survey of all 196 neonatal units in 2018 with a 95% response rate, however, demonstrated that only 18% of neonatal units used LISA regularly and only 2% performed LISA in the delivery suite (DS).^7^ This survey concluded that the use of LISA was uncommon in the UK despite being more commonly used in Europe. In the UK criteria for use and policies concerning premedication and technique of LISA varied.^7^ A subsequent UK‐based survey with 96% response rate in 2020 reported that 56% of units would consider LISA on the DS.^8^ This survey concluded that there was lack of training and national guidelines and that there was an urgent need for standardisation of practice and clear indications for LISA.^8^ A high level of competence for the LISA procedure, rescue intubation and CPAP application is required.^3^ Our aim, therefore, was to determine if the uptake of LISA and practice has changed compared to that reported in the previous UK survey.^7^, ^8^

## 2 METHODS

An online questionnaire was sent to all 191 neonatal units between June 2023 and May 2024. A web‐based survey link was sent to neonatal consultants of all included neonatal units in United Kingdom (total of 191 units). One consultant from each neonatal unit was randomly selected. A different consultant after 8 weeks was contacted if there was no response from the first consultant despite reminder emails. Responses were entered on an Excel sheet and reminder emails were sent to ‘non‐responders’ between June and Decemeber 2023. Further follow‐up was done by telephone to the medical team (middle‐grade doctor grade and above or alternatively to Advanced Neonatal Nurse Practitioners) in January 2024 for the non‐responders. The survey consisted of 16 multiple‐choice and open‐ended questions (Appendix S1). The questions were designed to be reflective of unit policy and not personal preference. Also, caution was exercised during the telephone survey that the answers were reflective of the unit practice. The clinicians were asked about the use of LISA in the delivery suite and the neonatal unit, the technique used, if premedication was given and if side effects had been experienced. The survey results were then compared with the previous published results.^7^ This project was registered as an audit with St George's University Hospitals NHS Foundation Trust (SGH) Audit department.

### 2.1 Statistical analysis

Differences between the two groups of survey results from 2018 and 2023–24 on the variables described in Table 1, level of neonatal unit, location where LISA performed, operator who performed, FiO_2_ criteria for LISA, sedation used, side effects post‐LISA were assessed for statistical significance using the McNemar test. IBM SPPS statistical software, V.29 (IBM Corporation, USA) was used.

**TABLE 1:** Survey results.

	2018 *n* (%)	2023–24 *n* (%)	*p* value
Responders—(yes/total)	187/196 (95%)	191/191 (100%)	
Level 1	45/46 (98%)	52/52 (100%)	
Level 2	84/88 (95%)	86/86 (100%)	
Level 3	58/62 (94%)	53/53 (100%)	
LISA performed (yes/total)			
Lisa performed	35/187 (19%)	134/191 (70%)	<0.001
Level 1	6/46 (13.3%)	22/52 (42%)	<0.001
Level 2	9/88 (10.7%)	62/86 (72%)	<0.001
Level 3	20/62 (34.5%)	50/53 (94%)	<0.001
LISA location (yes/total)			
NNU only	31/35 (89%)	103/134 (77%)	0.125
NNU + delivery suite	4/35 (2%)	31/134 (16%)	<0.001
LISA operator (yes/total)			
Consultant	32/35 (91.7%)	26/134 (19.4%)	0.031
Registrar/ANNP/equivalent	24 /35 (69.4%)	102/134 (76.1%)	<0.001
SHO/Equivalent	8/35 (25%)	6/134 (4.5%)	0.250
FiO_2_ criteria for LISA (yes/total)			
FiO_2_ > 0.3	22/35 (62.5%)	104/134 (77.6%)	<0.001
FiO_2_ > 0.4	16/35 (46.9%)	49/134 (35%)	<0.001
FiO_2_ > 0.5	3 /35 (9.4%)	22/134 (16.4%)	<0.001
Premedications/sedation (yes/total)			
Non‐pharmacological methods + Routine sedation	18/35 (51%)	45/134 (33.6%)	<0.001
Non‐pharmacological + sedation only if required	17/35 (49%)	89/134 (66.4%)	<0.001
Side effects (yes/total)	16/35 (45.7%)	65/134 (48.5%)	
Desaturation/hypoxia	5/35 (15.6%)	82/134 (61.3%)	<0.001
Bradycardia	6/35 (18.8%)	80/134 (59.6%)	<0.001
Surfactant reflux	5/35 (15.6%)	37/134 (27.4%)	<0.001
Pneumothorax	0 (0%)	1/134 (0.7%)	1.000

## 3 RESULTS

There was a 100% response rate from the 191 units neonatal units. Fifty‐three responders were neonatal intensive care units (NICU = level 3), 86 local neonatal units (LNU = level 2) and 52 special care baby units (SCBU = level 1). The number of neonatal units due to reconfiguration had reduced compared to the survey done in 2018 (191 vs. 196) (Table 1). Fourteen units reported plans to introduce LISA in the next year which would increase units using LISA to 148/191 (77.4%) from 134/191 (70%) actively using LISA in 2023–24. In the remaining 43 units, the predominant reason why LISA was not done in neonatal units was lack of experience/training (22/43, 51%) or not having a standardised practice/guideline (21/43, 49%). LISA in the delivery suite (DS) had increased from 4/35 (2%) in 2018 to 31/134 (16%) in 2024. The reasons why LISA was not performed in the DS were concerns regarding being an awake procedure 2/103 (2%), that further evidence of efficacy was required 10/103 (9.7%), lack of experience/training 28/103 (27%), practicalities/logistics 101/103 (98%) and not enough time to observe post‐procedure 2/103 (2%). The UK medical workforce usually consists of three or four tiers of doctors, consultants, middle grade (registrar or equivalent), senior house officer and/or foundation year doctor. Advanced nurse practitioners (ANNPs) work alongside the medical workforce in parallel or work instead of the middle‐grade/senior house officer depending on the years of experience and contract with the Trust. LISA was performed by more junior staff, registrars or equivalent, senior house officer or ANNPs (94%) in 2024 compared to more consultants performing LISA in 2018, (91.7% of units (*p* < 0.001). Sixty per cent (93/134) of units used Video laryngoscope (VDL) routinely for LISA, 4% used both direct and VDL and 27% (36/134) did not use VDL routinely for LISA in the current survey. Criteria for using LISA in the delivery suite or in the neonatal unit had lowered with more units offering LISA when an infant had an FiO_2_ more than 0.3 compared to more than 0.4 and 0.5 *p* < 0.001. Criteria for LISA in the DS in 2024 were an FiO_2_ > 0.3: 19/31 (61%), FiO_2_ > 0.4: 4/31 (13%), FiO_2_ > 0.5: 3/31 (10%), no documented criteria in5/31 (16%). Criteria for LISA in NNUs in 2024 were FiO_2_ > 0.3: 72/134 (53%), FiO_2_ > 0.4: 29/134 (22%), FiO_2_ > 0.5: 18/134 (13%), no documented criteria: 15/134 (12%). Based on gestational age (GA), for infants born <28 weeks, LISA was considered if FiO_2_ > 0.3, 60/134 (45%), FiO_2_ > 0.4, 16/134 (12%), FiO_2_ > 0.5, 7/134 (5%). For infants born ≥28 weeks FiO_2_ > 0.3, 57/134 (43%), FiO_2_ > 0.4, 27/134 (20%), FiO_2_ > 0.5, 10/134 (7%). For infants born at term (>37 weeks) FiO2 > 0.4 was considered in 3% (4/134) of units. There were no GA‐specific criteria in 52/134, and 39% of units and 3% (4/134) units were based on consultant decision. Fewer units were using routine sedation in 2024 (33.6% vs. 51%, *p* < 0.001) and more likely to use non‐pharmacological methods and use sedation only if required (66.4% vs. 49%, *p* < 0.001) in 2024 compared to 2018 (Table 1). Commonly used agents were atropine 32/134 (24%), fentanyl 56/134 (42%), morphine 7/134 (5.2%) and propofol 8/134 (6%). More side effects were reported in the 2024 survey compared to 2018. The most common side effects were bradycardia (59.6% vs. 18.8%, <0.001), desaturations/hypoxia (61.3% vs. 15.6%, *p* < 0.001), surfactant reflux into the oropharynx (27.4% vs. 15.6%, *p* < 0.001): there was only one reported pneumothorax in 2024 needing chest drain insertion following LISA.

## 4 DISCUSSION

We have demonstrated that LISA is more commonly performed in the UK in 2023 compared to 2018. The usage is much greater than in previously published European and UK surveys.^6^, ^7^, ^8^ Forty‐two percent of local neonatal units were using LISA, but the commonest reason for not using LISA was lack of experience and training. Similar observations were noted in the most recent survey published in a developing country^9^ and previously published UK surveys.^7^, ^8^ There is increased use of LISA in delivery suite (DS), and we have previously demonstrated that prematurely born infants who received LISA in the DS had comparable clinical outcomes to infants who received LISA on NNU.^10^ There are advantages of offering LISA in DS with reduced need for mechanical ventilation and costs of care.^11^ The predominant reason for the lack of use of LISA in DS was practicalities and logistics like location of delivery suite, space, availability of skilled staff to perform the procedure, lack of specific guideline, and VDL not readily available in DS were some of the reasons.

The oxygen threshold for the use of LISA in both the DS and NNUs had reduced to a percentage oxygen requirement of 30%. This threshold is in keeping with the recommendations from European Consensus Guidelines on the Management of Respiratory Distress syndrome^1^ which was based on observations of CPAP failure rates according to early postnatal oxygen requirements.^9^, ^10^ Moreover, change in the European consensus guidelines on the RDS guideline from considering LISA as alternative to INSURE for spontaneously breathing infants in 2016^11^ to LISA being the preferred technique in 2022^1^ when the FiO_2_ was above 0.3 when on non‐invasive ventilation (NIV) is the likely reason for the reduction in threshold for percentage of oxygen requirement.^1^, ^11^ There is varying oxygen requirement cut off criteria for administering LISA with some units using higher FiO_2_ criteria. A recent systematic review^12^ which included 58 RCTs inferred that surfactant administration may be considered in preterm infants born ≤30 weeks of gestational age requiring an FiO_2_ ≥ 40%. They concluded that there was insufficient evidence for the comparison of FiO_2_ thresholds: 30% versus 40%.^12^ The current survey indicates less use of sedation but more use of non‐pharmacological methods for analgesia such as swaddling, sucking on a dummy or sucrose. In a recent study of 153 LISA experts,^13^ 41% reported no use of pre‐procedure sedatives or analgesics, and 49% reported using fentanyl as a pre‐procedure treatment. On the contrary, 4% indicated no use of non‐pharmacological treatment. LISA may be uncomfortable, but in a metanalysis of one randomised control trial (RCT) (78 neonates), two observational studies (519 neonates), and 30 studies (2164 neonates) there was more apnoeic episodes post‐procedure requiring positive pressure ventilation if sedation was used, relative risk (RR), 95% confidence intervals (CIs): 3.13 (1.35; 7.24).^14^ In practice, the ease of the procedure seemed unaffected whether opiates, oral sucrose, or no sedation was used.^15^ Further clinical trials such as NON‐pharmacological Approach Less‐Invasive Surfactant Administration (NONA‐LISA, NCT05609877)^16^ and Premedication for Less‐Invasive Surfactant Administration Study (PRELISA, NCT05065424)^17^ trials are underway to assess benefits and risks of sedation for LISA.^16^, ^17^ Alternative methods of depositing adequate surfactant doses into the lung in a gentler way with equivalent efficacy would be ideal. Laryngeal masks can be used to administer surfactant in babies.^18^ Modern nebulisers are capable of aerosolising surfactant, but to date, studies on nebulisation have not convincingly shown any meaningful improvement in smaller infants who should benefit most.^19^ There were increased reports of side effects in the current survey compared to previous studies, it is not clear however if this was because more junior members of the team were performing LISA or if they were more likely to report side effects.

The main strength of our study is the 100% response rate from all neonatal units in the UK. Given that the uptake of LISA has increased over the years, the transition of LISA being offered in the DS seems likely to happen. Further research in this area would be beneficial. The likely reason for increased uptake of LISA in recent years compared to previous surveys is the availability of skilled staff to perform the procedure with improved training opportunities and unit‐specific guidelines. We appreciate some of the answers specifically relating to side effects of the procedure may reflect personal practice to some extent and may not be a reflection of the unit policy. This survey highlights the importance of training for clinicians where they plan to implement LISA. Both previous UK surveys published were of similar design to our current survey allowing comparison.

In conclusion, the uptake of LISA has increased in the United Kingdom compared to previous years. There are trends for more units using LISA in delivery suite and the use of video laryngoscope for LISA as the standard of practice. Lack of training and expertise continue to be the major limiting factors for neonatal units to perform LISA.

## AUTHOR CONTRIBUTIONS

**Sandeep Shetty:** Conceptualization; writing – original draft; methodology; validation; writing – review and editing; formal analysis; data curation; supervision. **Donna Tolentino:** Data curation. **Cheryl Lau:** Data curation; conceptualization. **Donovan Duffy:** Writing – review and editing. **Anne Greenough:** Conceptualization; validation; writing – review and editing; supervision.

## FUNDING INFORMATION

No funding required.

## CONFLICT OF INTEREST STATEMENT

Professor Greenough held a grant from Chiesi to examine the physiological effects of LISA in the delivery suite and has received funding from Chiesi as a speaker at webinars and conferences. Dr. Shetty has organised an Advanced neonatal respiratory course at St George's Hospital which was sponsored by Chiesi.

## ETHICS STATEMENT

None.

## CONSENT TO PARTICIPATE

None.

## CONSENT TO PUBLISH

None.

## Supporting information


Appendix S1.
