# Supplementary material for: Changes in practice of less‐invasive surfactant administration (LISA) in United Kingdom neonatal units
Source: Acta Paediatr. 2024 Oct 8;114(2):393–7. doi: 10.1111/apa.17446 (PMC11706752; doi:10.1111/apa.17446)
Supplement: Supplementary file 1 — Appendix S1. [file APA-114-393-s001.docx]

**Appendix: S1:**

**LISA Survey Questions**

1. Which neonatal unit do you work at?
2. Is your unit a:
   1. Neonatal intensive care unit
   2. Local neonatal unit
   3. Special care unit
3. Do you give LISA on the delivery suite?
   1. Yes
   2. No
4. If yes what are your criteria for who receives LISA on labour suite? Please tick all which apply
   1. Gestational age
      1. <24 weeks
      2. 24-26 weeks
      3. 26- 28 weeks
      4. 28-30 weeks
      5. 30-32 weeks
      6. >32 weeks
   2. FiO2 required to maintain saturations >88%
      1. >0.3
      2. >0.4
      3. >0.5
5. Is LISA given on the neonatal unit?
   1. Yes
   2. No
6. If yes what are your criteria for who receives LISA on the neonatal unit? Please tick all which apply
   1. Gestational age
      1. <24 weeks
      2. 24-26 weeks
      3. 26- 28 weeks
      4. 28-30 weeks
      5. 30-32 weeks
      6. >32 weeks
   2. FiO2 required to maintain saturations >88%
      1. >0.3
      2. >0.4
      3. >0.5
7. Who predominantly performs LISA?
   1. Consultant
   2. Registrar/equivalent
   3. SHO/equivalent
8. Do you use pre-medication or sedation prior to administration? If yes please select which you give
   1. None
   2. Swaddle
   3. Sucrose
   4. Fentanyl
   5. Atropine
   6. Opioids
   7. Ketamine
   8. Propofol
   9. Benzodiazepines
   10. Other
9. Do you undertake the procedure using video laryngoscopy?
   1. Yes
   2. No
10. Have you noticed any side effects? Tick all that apply
    1. None
    2. Surfactant reflux
    3. Hypoxia
    4. Bradycardia
11. What is the predominant reason for not using LISA in the delivery suite?
    1. Lack of experience and/or training
    2. Further evidence required
    3. Not part of standard practice
    4. Concerns around ‘awake’ procedure
    5. Practicalities/logistics
12. What is the predominant reason for not using LISA in the neonatal unit?
    1. Lack of experience and/or training
    2. Further evidence required
    3. Not part of standard practice
    4. Concerns around ‘awake’ procedure
    5. Practicalities/logistics
